# Supplementation of *Adiantum capillus-veneris* Modulates Alveolar Apoptosis under Hypoxia Condition in Wistar Rats Exposed to Exercise

**DOI:** 10.3390/medicina55070401

**Published:** 2019-07-23

**Authors:** Mehdi Yadegari, Maha Sellami, Simin Riahy, Shadmehr Mirdar, Gholamreza Hamidian, Ayoub Saeidi, Abderraouf Ben Abderrahman, Anthony C. Hackney, Hassane Zouhal

**Affiliations:** 1 Department of Exercise Physiology, Faculty of Physical Education and Sport Sciences, University of Mazandaran, Babolsar 4741613534, Iran; 2 Sport Science Program (SSP), College of Arts and Sciences (CAS), Qatar University, Doha 2713, Qatar; 3 Faculty of Aerospace Medicine and Subsurface, Army Medical University, Tehran 611/14185, Iran; 4 Department of Basic Sciences, Faculty of Veterinary Medicine, University of Tabriz, Tabriz 5166616471, Iran; 5 Department of Biological Sciences in Sport, Faculty of Sports Sciences and Health, Shahid Beheshti University, Tehran 1983969411, Iran; 6 ISSEP Ksar Said, University of Manouba, Tunis 2010, Tunisia; 7 Department of Exercise & Sport Science, University of North Carolina, Chapel Hill, NC 27599, USA; 8 Laboratoire M2S, University of Rennes, EA 1274, F-35000 Rennes, France

**Keywords:** respiratory disease, pathological apoptosis, Bax/Bcl-2 ratio, pneumocytes, exercise training

## Abstract

*Background and Objectives:* Several studies have reported that some conditions such as exercise and hypoxia induce DNA damage and dysfunction and apoptosis. Some plant foods contain numerous bioactive compounds and anti-inflammatory properties that can help fight DNA damage. Therefore, the current study evaluated the effect of supplementation of Adiantum capillus-veneris (ACV) extract on Bax/B-cell lymphoma 2 (Bcl-2) ratio apoptotic index and remodeling of pulmonary alveolar epithelial cells in lung tissue of healthy Wistar rats during stressful conditions (hypoxia). *Materials and Methods:* Twenty-seven Wistar male rats (four-week old, 72 ± 9 g) were randomly assigned into three groups: normoxic, sedentary, and not-supplemented (NG, *n* = 9); exercise and hypoxia and not-supplemented (HE, *n* = 9); and exercise and hypoxia and supplemented group (HS, *n* = 9). The NG remained sedentary in the normoxia environment for nine weeks. The HE group participated in a high-intensity (IT) program for six weeks, then remained sedentary in the hypoxia environment for three weeks. The low-pressure chamber simulated a ~2800 M altitude 24 h/d. HS participated in IT, then entered and remained sedentary in the hypoxia environment for three weeks, and they consumed 500 mg per kg of body weight ACV extract. *Results*: The Bax/Bcl-2 ratio of the HE group increased significantly (+50.27%, *p* ≤ 0.05), the average number of type I pneumocytes was reduced significantly (−18.85%, *p* ≤ 0.05), and the average number of type II pneumocytes was increased significantly (+14.69%, *p* ≤ 0.05). Also, after three weeks of consuming the ACV extract, the HS group in comparison with the HE group had their Bax/Bcl-2 ratio reduced significantly (−24.27%, *p* ≤ 0.05), the average number of type I pneumocytes increased significantly (+10.15%, *p* ≤ 0.05), and the average number of type II pneumocytes reduced significantly (−7.18%, *p* ≤ 0.05). *Conclusion:* The findings show that after three weeks of hypoxia following six weeks of high-intensity interval training in Wistar rats, the Bax/Bcl-2 ratio and the number of type II pneumocytes were increased and the number of type I pneumocytes was reduced significantly. These results strongly suggest that an apoptosis state was induced in the lung parenchyma, and consuming ACV extract modulated this state.

## 1. Introduction

Apoptosis, also called “programmed cell death”, is encoded by the genetic information within body cells and is the natural biochemical process through which they die and renew themselves. There are different pathways by which apoptosis occurs, among these, the death receptor mediated and mitochondrial-mediated pathways are the most critical, but the endoplasmic stress pathway also has some role in inducing the apoptosis process [1].

The mitochondrial-mediated pathway of apoptosis is regulated by the Bcl-2 (B-cell lymphoma 2) family of antiapoptotic (Bcl-2, Bcl-xl, Mcl-1) and proapoptotic proteins (Bax, Bad, and Bak). Specifically, Bcl-2 inhibits apoptosis by interacting and forming inactivating heterodimers with Bax/Bak [2]. Additionally, the Bax/Bcl-2 ratio is statistically correlated with age and tumor location [3,4]. Overexpression of Bcl-2 has been associated with drug resistance in hematologic malignancies, whereas a high Bax level is a good prognostic indicator in acute myeloid leukaemia (AML). This led some to suggest that the Bax/Bcl-2 ratio may be more important than either promoter alone in determining apoptosis [5].

The resistance to cell death, occurring upon disturbed balance of the Bax/Bcl-2 ratio, can be related to tumor cell invasion, cell metastatic [6]. Interestingly, the ratio Bax/Bcl-2 ratio showed a significant association with respiratory disease [7,8,9]. The respiratory airway and alveolar epithelial cells in particular are constantly exposed to a variety of external and internal alterations [10]. According to the authors of [11], epithelial cell apoptosis has been considered as the stimulator of numerous lung diseases. As previously discussed, several pathways can induce epithelial cell apoptosis, among these factors are stressful condition mediated by mitochondria such as exercise, hypoxia, drug consumption, radiation, infectious agents, and reactive oxygen species [11,12,13,14].

The epithelial surface of the alveoli is composed of alveolar type I and type II cells. Alveolar type I cells comprise 96% of the alveolar surface area. These cells are extremely thin, thus minimizing diffusion distance between the alveolar air space and pulmonary capillary blood. Type II cells are spherical pneumocytes, which comprise only 4% of the alveolar surface area, yet they constitute 60% of alveolar epithelial cells and 10%–15% of all lung cells. Four major functions have been attributed to alveolar type II cells: (1) synthesis and secretion of surfactant; (2) xenobiotic metabolism; (3) transepithelial movement of water; and (4) regeneration of the alveolar epithelium following lung injury. To this end, alveolar type II cells play important roles in normal pulmonary function and in the response of the lung to toxic compounds, which may cause lung damage [11,12].

However, so far, the effects of regular exercise on lung apoptosis and alveolar epithelial cells remodeling has not been well debated. Therefore, it would be interesting to clarify the extent to which exercise can regulate cell apoptosis in the lung, especially under hypoxic condition. In this context, it has been well demonstrated in most previous studies that chronic aerobic exercise could decrease apoptosis. In fact, the authros of [15] showed that aerobic exercise may reduce apoptosis on cardiomyocytes. It appears that the chronic aerobic exercise has a protective effect for the lung against apoptosis. In fact, moderate aerobic exercise can increase the endogenous antioxidants [16]. As known, these antioxidants play a major role in fighting the reactive oxygen species (ROS), which induces cellular apoptosis [17]. Despite this existing fact, emergent evidence suggested that interval training could induce better control of oxidative damage [9,18,19]. However, according to these previous studies, the cell damage is more related to exercise intensity. As such, understanding this fact would be of great interest in learning how the exercise-induced cell death and cell damage control occur at the same time.

As for exercise-induced cell death control, some natural food including antioxidants properties have been used as remedies in traditional medical systems for centuries, and are still being used as a source of bioactive compounds within pharmaceutical drug development [20]. One of most used plant base antioxidants is *Adiantum capillus-veneris*, used in traditional Chinese folk medicine for a variety of diseases. *Adiantum capillus-veneris* is traditionally used in the Unani system of medicine for the treatment of inflammatory diseases [21]. Chemical analysis of *Adiantum capillus-veneris* Linn reveals an array of compounds including triterpenes, flavonoids, phenylpropanoids, and carotenoids. On the basis of the common uses of this plant in traditional folk medicine, the present investigation was carried out to evaluate the anti-apoptotic and anti-remodeling effects of *Adiantum capillus-veneris* extract in the pulmonary alveolar of an animal engaged in intense exercise training (interval) and exposed to hypoxia [22].

It is well documented that athletes use hypoxia exposure to improve their physiological factors such as cardiovascular-repiratory adaption, specifically to improve oxygen carrying capacity. Along these lines, the reports of inflammatory and apoptosis complications with severe exercise and hypoxia exposure in the lung present a paradox for the athlete—hypoxia exposure can be helpful as well as harmful. Yet, mechanistic aspects of this situational paradox have not been well studied. Therefore, the present study aim was to investigate an answer to the question—if being in a hypoxia condition and performing high intensity exercise training would affect the Bax/Bcl2 ratio and pulmonary pneumocytes population, and what if any role *Adiantum capillus-veneris* extract supplementation would have on these adaptations.

To the best of our knowledge, this study is the first to evaluate alveolar apoptosis in animal species under hypoxic and normoxic condition and to see whether great exposure to high stimulus such as exercise as a regulator or cells death or additional antioxidant through supplementation will induce better control of the cell death in the lung.

## 2. Materials and Methods

### 2.1. Study Design

This study was carried out in accordance with the National Institutes of Health Guide for the care and use of laboratory animals. All experiments that involved animals were conducted according to the policy of the Iranian convention for the protection of vertebrate animals used for experimental and other scientific purpose and the protocol was approved by the Ethics committee of the Sciences, University of Mazandaran (UMZ) (number: 2316813 and date: 18 February 2016).

**Figure 1:**
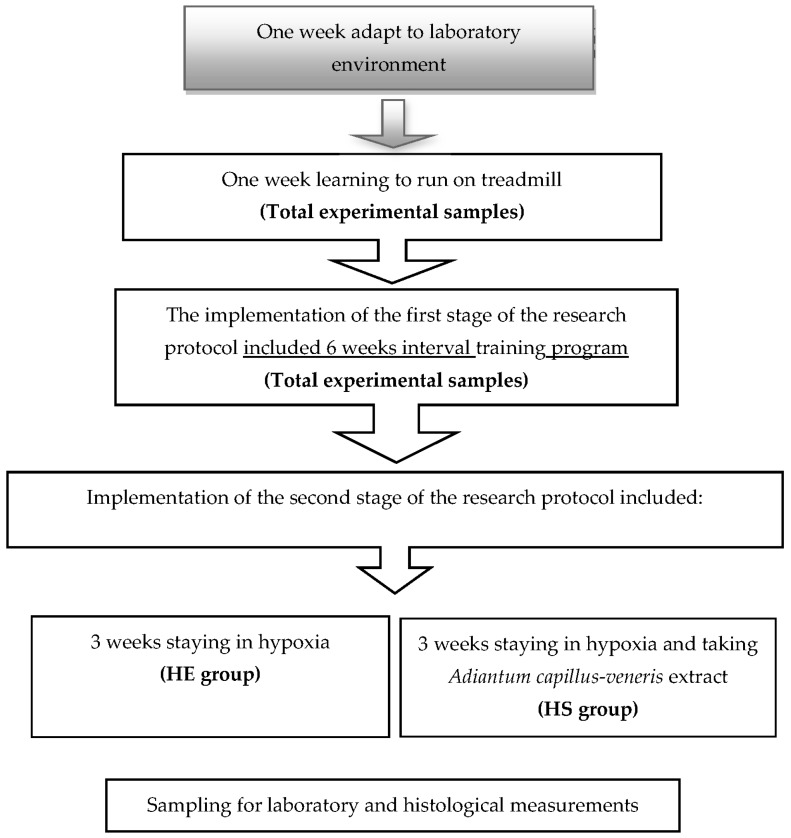
Experimental design. HS, exercise and hypoxia and supplemented; HE, exercise and hypoxia and not-supplemented.

Twenty-seven Wistar male rats (four weeks old, 72 ± 9 g weight) were acquired from Pasteur Institute (Amol, Mazandaran, Iran) and maintained in the central animal house of Faculty of Physical Education and Sport Science of University of Mazandaran. During the experiment, all animals were kept in standard polyester cage (46 L volume, two rats in each cage) in a room with standard temperature (22 ± 2 °C) and humidity (55% ± 5%) with a 12 h light/darkness cycle and free access to water and food. Animals were familiarized to environmental condition for one week and then were randomly assigned into normoxic, sedentary, and not-supplemented (NG, *n* = 9); exercise and hypoxia and not-supplemented (HE, *n* = 9); and exercise and hypoxia and supplemented (HS, *n* = 9) groups. The NG group remained sedentary in the normoxia environment for nine weeks. The HE group participated at interval training program for 6-week, then entered and remaining sedentary in the hypoxia environment for three weeks. The low-pressure chamber was artificially simulated altitude (~2800 m above sea level) and rats lived 24 h without training. The HS group participated in high-intensity (IT) for six weeks, then entered and remained sedentary in the hypoxia environment for three weeks, and they had consumed 500 mg per kg of body weight of *Adiantum capillus-veneris* (ACV) extract [23] in the hypoxic environment Figure 1.

### 2.2. Extraction of Adiantum capillus-veneris

A 500 g amount of *Adiantum capillus-veneris* fresh plant was shade dried, powdered, and extracted with ethanol for 6–8 h using soxhlet apparatus. The extraction process was done by packing 50 g of fine powder with Whatmann filter paper No.41 and placing of that in soxhlet apparatus along with solvent petroleum ether and followed by methanol. This extract contains both polar and non-polar phytocomponents [24] (Table 1).

### 2.3. Gaz chromatography-mass spectrometry (GC-MS) Analysis of Adiantum capillus veneris

A 5 mL extract was evaporated to dryness and re-dispersed into 2 mL methanol. The extracts were then subjected to GC-MS analysis. Chromatographic separation was carried out with instrument GC-MS-QP 2010 [Shimadzu] instrument with Db 30.0 column (0.25 μm diameter × 0.25 μm thickness). The oven temperature was programmed from 70 °C (isothermal for 5 min), with an increase of 10 °C/min, to 200 °C, then 5 °C/min to 280 °C, ending with a 35 min isothermal at 280 °C. Mass spectra were taken at 70 eV, with a scan interval of 0.5 s and scan range from 40–1000 m/z. Helium was used as carrier gas at 99.999% pressure with flow 1.0 mL/min and electronic pressure control on. Samples were dissolved in methanol and injected automatically [24].

#### ACV Extracts Contents

Table 1 shows the extract contains both polar and non-polar phytocomponents. It contains a significant level of n-Hexadecanoic acid (18.29%) and Gamma Sisterol (10.61%), as well as 9.25% of cis-vaccenic acid, which is an omega-7 fatty acid. Tetradecanoic acid, a saturated 14-carbon fatty acid, was found in 2.20% in the extract. In addition to Vitamin E, with 1.58% in the extract.

### 2.4. Exercise Training Protocol

In the present study, endurance interval training was used. At first, the animals were familiarized with rat treadmill apparatus, every day for five days. The experimental groups (HE and HS) were trained for six weeks using the same training protocol. In each training session, rats completed the 10 repetition of 1 min activity, with 2 min active rest between sessions. Work to rest ratio was 1:2 and total activity time was 30 min, 5 d/w. The running velocity began with 25 m/min and gradually reached to 70 m/min, as recommended in prior research [25]. These procedures are depicted in the procedure design overview (Table 2).

### 2.5. Tissue Sampling

For microscopical studies, the whole of the left lung was removed from the animals and fixed in 10% neutral buffered formalin. After tissue processing including dehydration in graded alcohol series, clearing in xylene, and impregnation in paraffin wax, the lung was embedded in a paraffin block according to an isotropic uniformly random (IUR) protocol. Each block was cut into four 20 µm thick serial sections and then four 5 µm thin serial sections using a rotary microtome. This process was continued to the end of the tissue within each block. Sections were collected on slides and were stained with hematoxylin–eosin (H&E). Systematic random sampling protocol was performed and the first section was chosen randomly. Finally, 20 to 25 thick sections and 5 thin sections were selected from each block for stereological and immunohistochemical analysis [26].

### 2.6. Immunohistochemical Analysis

**Figure 2:**
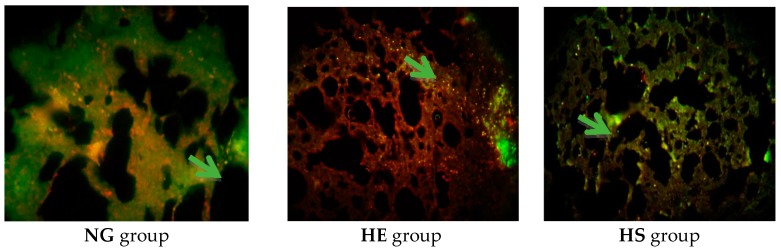
Expression of B-cell lymphoma 2 (Bcl-2) protein in lung tissue. Bcl-2 secondary antibody is connected to the FITC color and cell nuclei are stained with color PI. Differential staining with hematoxylin and magnification 200×. Green color (arrows) in the context is the sign of Bcl-2 expression. NG, normoxic, sedentary, and not-supplemented.

**Figure 3:**
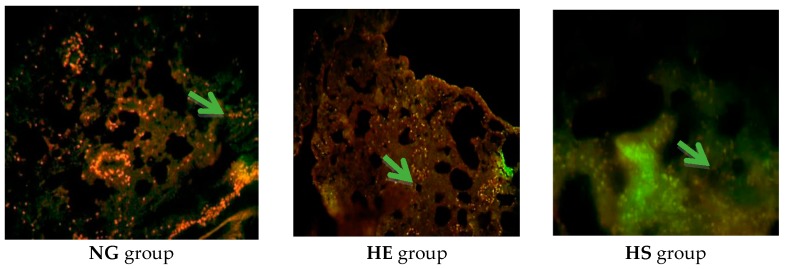
Expression of Bax protein in lung tissue. Bcl-2 secondary antibody is connected to the FITC color and cell nuclei are stained with color PI. Differential staining with hematoxylin and magnification 200×. Green color (arrows) in the context is the sign of Bcl-2 expression.

For immunohistochemistry, tissue sections were deparafinized in xylen, rehydrated in ethanol, and washed in PBST (PBS containing 0.1% Tween-20) following heat induced epitope retrieval with sodium citrate buffer (10 mM, pH 6.0) for 10 min. After three washes with PBST, sections were blocked with 10% normal goat serum in PBS containing 0.03% Triton X-100 for 3 h and then incubated with the appropriate primary antibody at a dilution 1:500–1:1000 for 24–48 h at 4 °C. The primary antibodies used were as follows: mouse anti-Bax antibody (Abcam USA, Cambridge, MA, USA, code: 044-436) and mouse anti-Bcl2 antibody (Millipore USA, Burlington, MA, USA, code: 69643). After secondary antibody treatment, the sections were washed twice for 5 min with PBST, then incubated for 5–10 min with DAPI (40,6-diamidino-2-phenylindole; 1:1000, from 2 mg/mL stock), and three additional wash steps of 10 min were performed with PBST and mounted with the anti-fade kit (Invitrogen, Carlsbad, CA, USA). Bright field microscopy was performed for visualization and images were captured with a digital camera attached to the microscope (Olympus BX 51TF) at different magnifications (Figure 2 and Figure 3). Separate red and blue filtered images were digitally combined to produce composite images. An experimenter blind to each condition used Image J (NIH, Bethesda, MA, USA) to calculate the fluorescence intensity or pixel value of twelve sections for each animal, which were averaged to get the value of one animal. The average pixel value of the negative control for each group was subtracted from the value of the respective experimental data [27].

### 2.7. Stereological Analysis

**Figure 4:**
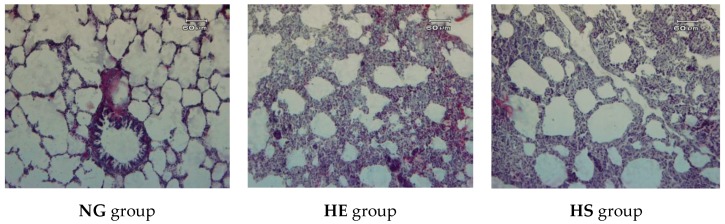
The histological structure of lung tissue in experimental groups (stained with hematoxylin-eosin, magnification, 200×). Parenchyma structure of **NG** group is normal, while in the **HE** group, increased emphysema and interstitial tissue is clearly visible. The number of alveoli and respiratory surface decreased in **HE** group. These changes dropped in the **HS** group.

In this study, we aimed to develop an efficient and practical design-based combination of stereological tools for the estimation of the pneumocytes number in lung of rat. Stereological studies were carried out strictly under blind condition with the optical fractionator for estimating number of type II and type I pneumocytes by version 9 stereo-investigator system (MBF Bioscience, Micro Bright Field, Inc., Germany, Hanover). This system consists of a standard microscope, a motorized stage, digital closed-circuit camera, and a software application Figure 4. To estimate the total number of alveolar pneumocytes, the 20 µm thick section and a high numerical aperture oil immersion lens were used.

By means of the stereological software, an unbiased counting frame was superimposed on lung sections. The number of type I and II pneumocytes was estimated using the optical dissector method and the following formula:

(1) $$N_{V\;(\mathrm{cell}/\mathrm{ref})}\;=\;\frac{\sum Q^{-}}{\sum A\times h},$$

where ∑*Q*^−^ is the number of cells coming into focus in the dissector height, ∑*A* is the total area of the unbiased counting frame in all microscopic fields, and *h* is the height of dissector. The total number of cells was estimated by multiplying the numerical density (*N*_v_) by the total volume of the lung [26].

### 2.8. Statistical Analysis

Descriptive statistics were used to determine the mean and standard deviations of the groups. The Kolmogorov–Smirnov test (k–s) was used to assess data distribution normality. One-way analysis of variance (ANOVA) with the Bonferroni post-hoc test was used to compare group means. All calculations were performed using SPSS 21.0 (Version 21; SPSS, Chicago, IL, USA) software and significance was set at *p* ≤ 0.05.

## 3. Results

### 3.1. Bax/Bcl-2 Results

**Figure 5:**
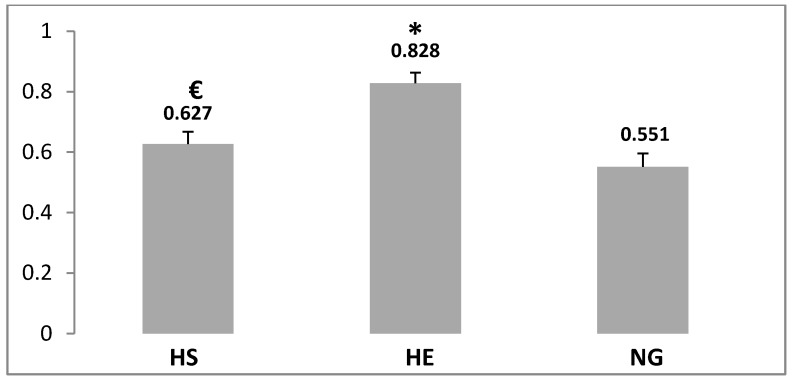
Analysis of Bax/Bcl-2 ratio (Pixle/um2) levels in the NG, HE, and HS groups. Data are means ± SEM. * Significant difference compared with NG group (*p* ≤ 0.05). **€**: Significant difference compared with HE group (*p* ≤ 0.05).

Statistical analysis showed that after six weeks of interval training and three weeks of hypoxia, Bax/Bcl-2 ratio increased significantly in the HE group compared with the other groups (50.27%, *p* ≤ 0.05, Figure 5). Bax/Bcl-2 ratio reduced significantly (24.27%, *p* ≤ 0.05, Figure 1) in the HS group compared with the HE group (*p* ≤ 0.05).

### 3.2. Pneumocytes Number in Response to Intervention

**Figure 6:**
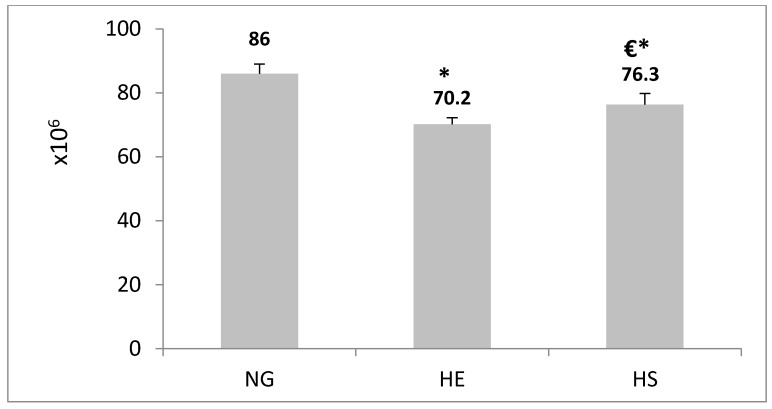
Analysis of average number of type I pneumocytes (×10^6^) in the NG, HE, and HS groups. Data reported are means ± SEM. * Significant difference compared with NG group (*p* ≤ 0.05). **€**: Significant difference compared with HE group (*p* ≤ 0.05).

Figure 6 shows the average number of type I pneumocytes was decreased significantly in the HE group compared with the NG group (18.85%, *p* ≤ 0.05). Also, after an extraction period, the number of type I pneumocytes increased significantly in the HS group compared with the HE group (9%, *p* ≤ 0.05).

**Figure 7:**
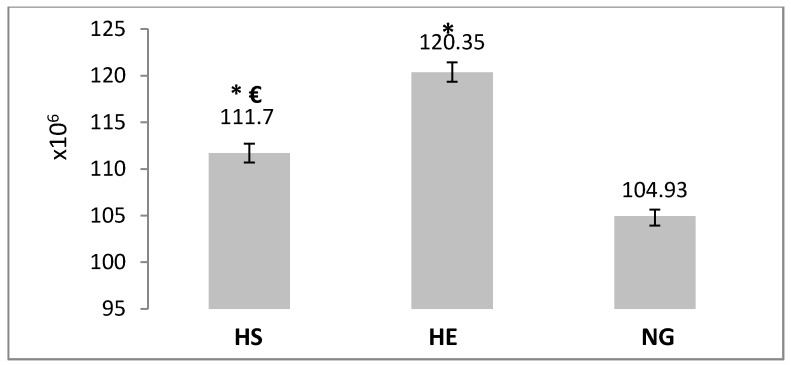
Analysis of average number of type II pneumocytes (×10^6^) in the NG, HE, and HS groups. Data are mean ± SEM. * Significant difference compared with NG group (*p* ≤ 0.05). **€**: Significant difference compared with HE group (*p* ≤ 0.05).

In addition, the average number of type II pneumocytes was increased significantly (14.69%, *p* ≤ 0.05, Figure 7). Also, after three weeks of consuming the *Adiantum capillus-veneris* extract in the hypoxia environment (HS group), in comparison with the HE group, the average number of type I pneumocytes was increased significantly (10.15%, *p* ≤ 0.05, Figure 6), and the average number of type II pneumocytes was reduced significantly (7.18%, *p* ≤ 0.05, Figure 7).

## 4. Discussion

Studies have reported that intense exercise and hypoxia induced DNA damage and have questioned a possible link to apoptosis. The aim of this study was to evaluate the effects of interval training and hypoxia on Bax/Bcl2 ratio apoptotic index and remodeling of pulmonary alveolar epithelial cells in lung tissue of healthy Wistar rats.

The major findings of this study showed that (1) after three weeks of hypoxia following six weeks of interval exercise training, the Bax/Bcl-2 ratio and the number of type II pneumocytes was increased and the number of type I pneumocytes was reduced significantly. Furthermore, after three weeks consuming of *Adiantum capillus-veneris* extract in the hypoxia environment, compared with the hypoxia alone group, Bax/Bcl-2 ratio reduced, the average number of type I pneumocytes was increased, and the average number of type II pneumocytes was reduced.

Limited research has been done related to hypoxia effects on apoptotic indicators in the trained samples lung tissue and existing knowledge is ambiguous [13]. After six weeks of high intensity interval training (HIIT), the expression of Bax and Bcl2 proteins was reported to be increased in the lungs [28,29]. Apoptosis was also evident immediately after a run to exhaustion and continued to be detectable 24 h post exercise [28]. Furthermore, the induction of apoptosis appeared to be mediated by glucocorticoid receptor activation as RU-486 (also known as mifepristone and used as an anti-progestin), a potent glucocorticoid receptor antagonist, decreased DNA fragmentation in the rats that experienced mild physical stress compared with controls [28].

On the other hand, it is well known that hypoxia can lead to cellular injury and death. Only recently, however, it has been appreciated that hypoxia can induce apoptosis as well as necrotic cell death; that is, studies report that hypoxia induces apoptosis via a mitochondrial pathway involving the release of cytochrome-c and subsequent caspase activation [29,30].

On the basis of our data, it is possible to postulate that intensive periodic exercise before exposure to a hypoxic environment would increase the onset and appearance of apoptosis in the lung. It is reported that athletes, especially endurance athletes who are engaged in intense exercise, such as daily repeated long runs, experience damage and increased inflammation in the respiratory mucosa. Intense exercise by creating a temporary hypoxia and metabolic tissue reperfusion during rest would provide for conditions of accelerated free radical formation. Oxidative stress damage caused by reactive oxygen species (ROS) production can cause irreversible damage to the lung tissue; and the intensity and duration of physical activity have an important effect on the production of such free radicals [25,29].

Another important regulator of the mitochondrial apoptotic pathway in hypoxia is the proapoptotic Bcl-2 family member Bax. Translocation of Bax to the mitochondria triggers cytochrome c release in many cells during and after hypoxia [31]. In addition, levels of Bcl-2, which prevent cytochrome c release, are reduced by hypoxia in several systems [28,29]. These processes occur both during hypoxia as well as during re-oxygenation. More proximal regulators of Bax and cytochrome c release during hypoxia may include hypoxia-inducible factor-1a (HIF-1a) and p53. In the present study, we report increased levels of the apoptotic Bax/Bcl-2 ratio in pulmonary alveolar, thus it seems that after the interval training program and hypoxia environment exposure, the apoptosis state was induced in lung parenchyma.

Moreover, in this study, the use of ACV extract in the hypoxia group reduced the Bax/ Bcl2 ratio and reduced the population of epithelial cells to their normal levels. ACV plants extracts are widely used for treating the respiratory system relative to dyspnea, asthma, and chest pain. It has been also used for gastrointestinal problems such as jaundice, diarrhea, and abdominal pain. Interestingly, recent studies have investigated ACV anti-inflammatory properties [20]. In fact, according to Yuan et al., the plant extract suppressed PGE2 (Prostaglandin), IL-6 (Interleukin 6), and TNF (Tumor necrosis factor) release with an IC50 less than 50 μg/mL. These researchers also found that the ACV could normalize the lipopolysaccharide-induced increase of spleen index in rats. It normalizes also the NF-κB (Nuclear factor kappa-light-chain-enhancer of activated B cells) and p38 activations in CD1 mice [20]. The identification of Gamma-sitosterol, a substance that opposes oxidation or inhibits reactions brought about by dioxygen or peroxides, may explain the reduction of Bax/Bcl2 ratio and reduction of DNA damage. The presence of Gamma-sitosterol in the ACV extract has been proven, which may be a reduction mechanism in the Bax/Bcl2 ratio of the lung under hypoxia conditions [21,22]. ACV extract may thus be use as a medicinal plant; that is, as a powerful anti-inflammatory agent and a suppressor of apoptosis alveolar.

Furthermore, histological changes of lung tissue after exposure to hypoxia in trained rats showed the number of type I pneumocytes were decreased, while the number of type II pneumocytes was increased. Because type I pneumocytes play the main role in the structure of the alveolus and make up more than 90% of its surface, it seems that these changes lead to a reduction of respiratory surface and a state similar to pulmonary fibrosis [32,33]. Hence, interval exercise training and hypoxia via increased pulmonary ventilation leads to increase strain on the airways and tearing of the respiratory epithelium, resulting in inflammation and stretching the epithelium wall. Proliferation of type II pneumocytes has been linked to a repair process during the early phase of acute lung injury, and it persists for a variable period. The mechanisms responsible for their dissolution and/or disappearance are not known, but we speculate that it may be partly due to apoptosis. In chronic interstitial pneumonia caused by hypoxia, type II pneumocytes proliferate continuously, although to a much lesser degree than in the early phase of acute lung injury, and are minimally apoptotic [34,35].

This hypoxia induced effect was comparable to that provoked by angiotensin II, a potent inducer of cell apoptosis, and may be linked to the development of lung fibrosis [36]. The hypoxic signal transduction included HIF-1α (Hypoxia-inducible factor 1-alpha) stabilization, nuclear translocation, and HRE-dependent gene (hypoxia-responsive elements) activation with the proapoptotic protein Bnip3L as a major target gene. Transient transfection with HIF-1α cDNA enhanced the hypoxia-induced apoptosis. This hypoxia-induced cell death may impact upon alveolar epithelium homeostasis under conditions of chronic alveolar oxygen deprivation, with involvement of the HIF-1 signaling pathway and Bnip3L as major downstream effector.

The increase in the of number type I pneumocytes and decrease in number of type II pneumocytes with a ACV supplement may indicate the growth and anti-apoptotic properties of this medicinal plant. In confirmation of these assumptions, the ACV extract was able to inhibit the production of PGE2 due to LPS (Lipopolysaccharides) function, which could also inhibit the production of TNF-α and IL-6 in monocyte/macrophage activity [20]. An important part of these inhibitory effects is NF-κB inactivation [16].

Hypoxia also reinforces expression of pro-inflammatory interleukins and reactive oxygen species [37]. Inflammatory and oxidative factors can have a strong role in inducing apoptosis [38,39].

Regarding the findings of the present study, it seems that intense exercise training reduces the ability of the lung to cope with hypoxia complications and causes the tissue to be exposed to cell death and defective remodeling. For this reason, in athletes who perform intense exercise, it may be necessary to reduce exercise load before going to altitude and staying there for lengthy periods [28]. Although, another alternative method to modulate the effects of severe exercise training and hypoxia on the lungs may be the use of antioxidant and anti-inflammatory supplements, such as ACV [20]. Hence, nutritional support and supplementation for athletes may be necessary as it appears the antioxidant and anti-apoptotic systems of the lung are not able to maintain tissue homeostasis and protect it in situations in which extremely elevated ventilation occurs.

## 5. Conclusions

The findings of this study in general showed that after the interval training program and hypoxia environment exposure, the apoptosis state was induced in lung parenchyma, and may challenge the physiology and morphology of this tissue. Apoptotic cell death induced by hypoxia after exercise may be a normal process used to remove partially damaged cells that are produced after training. Interestingly, consuming *Adiantum capillus-veneris* extract may modulate this state by reducing the Bax/Bcl-2 ration and increasing the pneumocytes I in the population of rats. Future research is needed to determine the molecular signals enhancing alveolar apoptosis in order to develop other potential therapeutic protection and interventions to protect pulmonary epithelial cells. Accurate recognition of the pathways involved in hypoxia-dependent apoptosis and discovery of the main anti-apoptosis elements in ACV extract can be applied targets in future studies.

## Figures and Tables

**Table 1:** The main components of the *Adiantum capillus-veneris* measured by GC-MS (Gaz chromatography-mass spectrometry) analysis.

Components	The Area under the Peak (%)	Retention Time (min)
5-(7A-isopropenyl-4,5-dimethyloctahydro-1H-inden-4-yl	24.49	28.871
n-Hexadecanoic acid	18.29	17.349
Gamma-Sistosterol	10.61	28.101
Cis-vaccenic acid	9.25	19.015
5-(7A-isopropenyl-4,5-dimethyl-octahydroinden-4-YL)-3methyl-pent-2-EI	2.63	30.443
Tetradecanoic acid	2.20	15.184
Phenanthrene, 9-dodecyltetradecahydro	2.15	29.349
Squalence	2.13	24.166
4-(2,6,6-trimethyl-1-cyclohexen-1-YL)-3-penten-2-one	1.92	28.101
cis-9-Hexadecenoic acid	1.78	17.050
2-Azapentane-1,5-dione, 4-methyl-1,5-diphenyl-3-(p-tolyl)-	1.74	29.758
Hexaethylene glycol monododecyl ether	1.60	24.734
Vitamin E	1.58	26.411
**Total**	80.37	

**Table 2:** Sprint-interval training program performed by the training rats. Treadmill speed, resting period, sets, and frequency varied according to the rats’ familiarity and fitness level.

Week	Familiarization	1	2	3	4	5	6
**Age (week)**	6	7	8	9	10	11	12
**Treadmill speed (m·min^−1^)**	10–25	25–35	35–45	45–55	55–65	65–70	65–70
**Sprint duration (min)**	1	1	1	1	1	1	1
**Resting period (min)**	2	2	2	2	2	2	2
**Sets (N·day^−1^)**	10	10	10	10	10	10	10
**Frequency (days·week^−1^)**	5	5	5	5	5	5	5
